# Sodium Intake Among U.S. Adults — 26 States, the District of Columbia, and Puerto Rico, 2013

**Published:** 2015-07-03

**Authors:** Jing Fang, Mary E. Cogswell, Soyoun Park, Sandra L. Jackson, Erika C. Odom

**Affiliations:** 1 Division for Heart Disease and Stroke Prevention, National Center for Chronic Disease Prevention and Health Promotion, CDC

Excess sodium intake is a major risk factor for hypertension, and subsequently, heart disease and stroke, the first and fifth leading causes of U.S. deaths, respectively (1). During 2011–2012, the average daily sodium intake among U.S. adults was estimated to be 3,592 mg (2), above the *Healthy People 2020* target of 2,300 mg (3). To support strategies to reduce dietary sodium intake, 2013 Behavioral Risk Factor Surveillance System (BRFSS) data from states and territories that implemented the new sodium-related behavior module were assessed. Across 26 states, the District of Columbia (DC), and Puerto Rico, 39%–73% of adults reported taking action (i.e., watching or reducing sodium intake) (median = 51%), and 14%–41% reported receiving advice from a health professional to reduce sodium intake (median = 22%). Compared with adults without hypertension, a higher percentage of adults with self-reported hypertension reported taking action and receiving advice to reduce sodium intake. For states that implemented the module, these results can serve as a baseline to monitor the effects of programs designed to reduce sodium intake.

BRFSS is an annual, random-digit–dialed telephone survey representative of noninstitutionalized, civilian adults aged ≥18 years in each U.S. state and territory. Detailed information on the survey is available at http://www.cdc.gov/brfss. In 2013, 26 states, DC, and Puerto Rico implemented the new, optional sodium-related behavior module. The median American Association of Public Opinion Research location-specific response rate was 48.1% (range = 31.1%–60.3%) (4).

Taking action to reduce sodium intake was defined by a “yes” response to the question, “Are you currently watching or reducing your sodium or salt intake?” Receiving health professional advice to reduce sodium intake was defined by a “yes” response to the question, “Has a doctor or other health professional ever advised you to reduce sodium or salt intake?” Self-reported hypertension was defined by a “yes” response to the question, “Have you ever been told by a doctor, nurse, or other health professional that you have high blood pressure?” The percentage of respondents taking action or receiving advice to reduce sodium intake was estimated for each state overall and by self-reported hypertension status. All estimates were age-standardized using the 2000 U.S. standard projected population. States were categorized in quartiles based on age-standardized proportions of respondents reporting taking action to reduce sodium intake and on proportions reporting having received advice to reduce sodium intake.

A total of 185,463 participants answered questions from the optional sodium module. After excluding 5,396 participants with missing information on key variables, 180,067 participants were included. State sample sizes ranged from 3,332 (Massachusetts) to 12,363 (Minnesota). The proportion of respondents who reported taking action to reduce sodium intake ranged from 38.7% (Utah) to 73.4% (Puerto Rico), with a median of 50.6% (Table 1). Across all participating locations, a higher proportion of participants with hypertension reported taking action to reduce sodium intake compared with those without hypertension (p<0.001 for all comparisons) (Table 1).

The proportion of participants who reported receiving advice from a health professional to reduce sodium intake ranged from 13.5% (Minnesota) to 41.4% (Puerto Rico), with a median of 21.1%. Across all locations, a higher proportion of participants with hypertension reported receiving health professional advice to reduce sodium intake compared with those without hypertension (p<0.001 for all comparisons) (Table 2).

Although only 10 of the 28 survey areas were in the Southern U.S. Census Region,* most of the survey areas with the highest proportions of respondents reporting taking action to reduce sodium intake and most of those with the highest proportion of respondents reporting having received advice from a health professional to reduce sodium intake were in the South. Eight of 10 states in the South were in the top two quartiles for taking action; the two that were not in the top two quartiles were West Virginia and Kentucky (Figure 1). All 10 states in the South were in the top two quartiles for receiving advice. The other four survey areas in the top half were Connecticut, New Jersey, Hawaii, and Puerto Rico (Figure 2).

## Discussion

In 2013, across 26 states, DC, and Puerto Rico, the proportion of respondents who reported both taking action and receiving advice to reduce sodium intake varied, with generally higher proportions in states in the Southern U.S. Census Region, Missouri, some states in the Northeastern U.S. Census Region, and Puerto Rico. Overall, approximately half of U.S. adults in participating states and territories reported taking action to reduce sodium intake, and about one in five reported receiving advice from a health professional to reduce sodium intake. Respondents with self-reported hypertension were more likely to take action and receive advice to reduce sodium intake than those without. However, among adults with self-reported hypertension, 20% (Puerto Rico) to 50% (Utah) did not report taking action to reduce sodium intake. In all but four locations (DC, Kentucky, New Jersey, and Puerto Rico), less than half of respondents reported receiving advice to reduce sodium intake. Among adults without hypertension, most did not report taking action to reduce sodium intake, and an even smaller proportion reported receiving professional advice to reduced sodium. These findings suggest an opportunity for promoting strategies to reduce sodium consumption among all adults, with and without hypertension.

This is the first report with state-level estimates of sodium intake behavior among the general population. The geographic pattern of the prevalence of taking action or receiving advice to reduce sodium intake appears to roughly correspond with the pattern of the prevalence of self-reported hypertension (5). BRFSS 2009 data indicate the prevalence of self-reported hypertension is generally higher in the Southern U.S. Census Region, plus Indiana, Michigan, Missouri, Ohio, Pennsylvania, and Rhode Island. A possible explanation for the higher prevalence of taking action and receiving health professional advice to reduce sodium intake in Connecticut and New Jersey could be proximity to New York City’s (NYC) media campaign promoting sodium reduction and other NYC and state programs aimed at reducing sodium intake. For example, in April 2013, NYC launched a communication campaign for consumers to purchase lower-sodium foods.†

The finding that Puerto Rico had the highest percentage of respondents both taking action and receiving advice for sodium reduction is new. The high percentages might be related to high hypertension prevalence. Based on 2013 BRFSS data, the prevalence of self-reported hypertension in Puerto Rico was 42.3%, whereas the national prevalence was 31.4% (6).

The findings in this report are subject to at least four limitations. First, BRFSS data are self-reported and subject to recall and social desirability bias, which might overestimate or underestimate prevalence. Second, the methods used by participants to watch or reduce sodium intake were not assessed. Third, these results are not generalizable to the entire United States. Although CDC encouraged states to use the module to assess the sodium-related behavior, the reasons individual states chose to use the module is unknown. Finally, response bias is possible because BRFSS response rates were <50%. Despite these limitations, this report is the first to provide multistate data on sodium-reduction behavior among all BRFSS respondents.

The data in this report highlight the opportunity to increase the proportion of health care professionals who advise their patients to reduce sodium intake and the proportion of U.S. adults who take action to reduce sodium intake. During 2011–2012, approximately 48% of hypertension among U.S. adults was uncontrolled (7). From 2010 to 2030, total direct medical costs of cardiovascular disease are projected to triple, increasing from $273 billion to $818 billion (in 2008 U.S. dollars) (8). Reducing sodium intake by 1,200 mg daily is projected to save $18 billion in health care costs yearly (9). Health care professionals can make a difference by recommending healthy dietary patterns, such as the Dietary Approaches to Stop Hypertension (10). By expanding the use of the sodium-related behavior module, states can enhance the ability to evaluate the effects of sodium-reduction campaigns.

**Summary**

What is already known on this topic?

National surveillance data show that current sodium intake in the United States is substantially higher than recommended. Excess sodium intake is an important risk factor for hypertension.

What is added by this report?

In 2013, among 26 states, the District of Columbia, and Puerto Rico, the median prevalence of taking action to reduce sodium intake was 51%, ranging from 39% to 73%. The median prevalence of receiving health professional advice to reduce sodium intake was 22%, ranging from 14% to 41%. Although action and advice were higher among hypertensive participants across locations, 20%–50% did not report taking action, and 38%–68% reported not receiving advice to reduce sodium intake.

What are the implications for public health practice?

These data highlight the opportunity to increase the proportion of health professionals who advise their patients to reduce sodium intake and the proportion of U.S. adults who take action to reduce sodium intake.

## Figures and Tables

**FIGURE 1:**
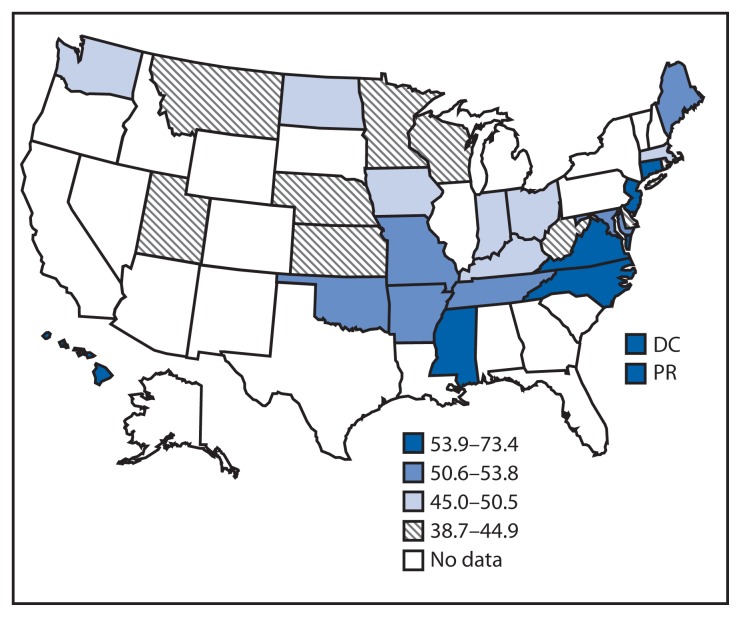
Age-adjusted percentage of adults aged ≥18 years who reported taking action to reduce their dietary sodium intake — 26 states, the District of Columbia, and Puerto Rico, Behavioral Risk Factor Surveillance System, 2013

**FIGURE 2:**
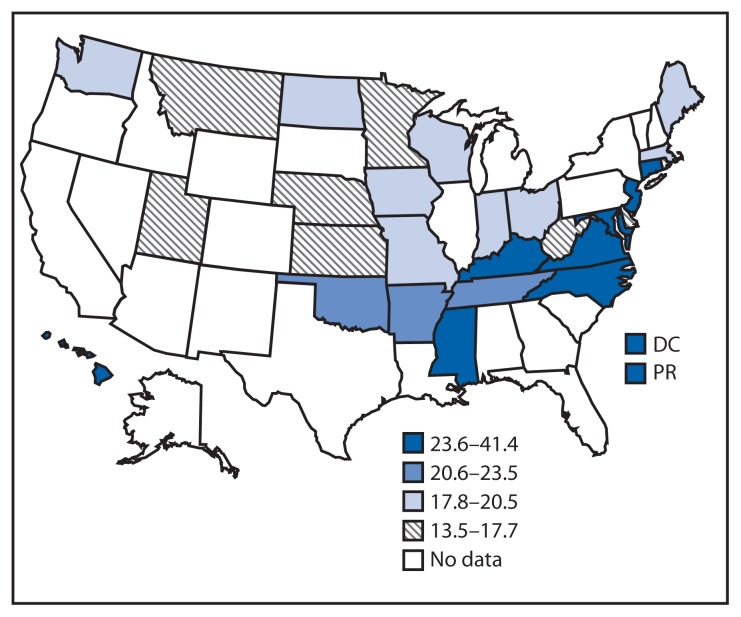
Age-adjusted percentage of adults aged ≥18 years who reported being advised by a health professional to reduce dietary sodium intake — 26 states, the District of Columbia, and Puerto Rico, Behavioral Risk Factor Surveillance System, 2013

**TABLE 1:** Age-adjusted percentage of adults aged ≥18 years who reported taking action to reduce their dietary sodium intake, by hypertension status — 26 states, the District of Columbia, and Puerto Rico, Behavioral Risk Factor Surveillance System, 2013

	Overall			Self-reported hypertension			No self-reported hypertension		
			
State/Area	No.	%	(95% CI)	No.	%	(95% CI)	No.	%	(95% CI)
Arkansas	4,469	52.6	(50.3–54.9)	2,231	62.9	(57.6–67.8)	2,238	47.1	(44.4–49.8)
Connecticut	6,547	50.7	(48.8–52.6)	2,589	67.7	(62.6–72.4)	3,958	44.0	(41.9–46.2)
DC	3,990	54.8	(52.2–57.4)	1,623	70.8	(63.7–77.0)	2,367	47.8	(44.7–50.8)
Hawaii	6,992	55.8	(54.0–57.5)	2,204	63.2	(57.9–68.2)	4,788	52.8	(50.8–54.8)
Indiana	4,362	45.2	(43.3–47.3)	1,904	57.0	(51.9–62.0)	2,458	39.8	(37.5–42.1)
Iowa	7,210	45.5	(43.9–47.1)	2,889	57.7	(53.1–62.2)	4,321	40.6	(38.9–42.4)
Kansas	10,947	43.3	(42.1–44.4)	4,455	55.9	(52.5–59.1)	6,492	37.7	(36.4–39.1)
Kentucky	9,704	50.5	(48.9–52.1)	4,717	72.4	(69.0–75.6)	4,987	39.2	(37.2–41.1)
Maine	4,496	52.3	(50.3–54.4)	1,807	67.7	(61.5–73.3)	2,689	47.6	(45.2–49.9)
Maryland	11,473	52.2	(50.7–53.7)	4,907	63.7	(60.1–67.2)	6,566	46.4	(44.6–48.1)
Massachusetts	3,332	49.8	(46.5–53.2)	1,343	61.7	(50.9–71.5)	1,989	45.4	(41.7–49.1)
Minnesota	12,363	40.7	(39.2–42.3)	4,256	52.9	(48.9–56.8)	8,107	35.2	(33.3–37.1)
Mississippi	6,628	56.3	(54.4–58.1)	3,514	66.1	(61.8–70.1)	3,114	48.6	(46.2–50.9)
Missouri	5,478	51.2	(48.9–53.5)	2,527	58.6	(53.3–63.6)	2,951	46.9	(44.2–49.6)
Montana	4,517	44.9	(42.9–46.9)	1,706	55.3	(50.0–60.6)	2,811	40.0	(37.7–42.3)
Nebraska	7,667	44.8	(43.0–46.6)	3,095	56.2	(51.4–61.0)	4,572	39.5	(37.4–41.5)
New Jersey	3,700	59.3	(56.8–61.8)	1,365	71.9	(63.6–79.0)	2,335	54.5	(51.5–57.3)
North Carolina	3,824	58.2	(56.1–60.4)	1,749	70.8	(65.2–75.8)	2,075	53.2	(50.6–55.8)
North Dakota	6,932	45.7	(44.0–47.3)	2,583	60.7	(55.2–65.9)	4,349	40.4	(38.5–42.3)
Ohio	7,138	46.0	(44.3–47.7)	3,078	56.8	(52.5–61.0)	4,060	40.0	(38.0–42.1)
Oklahoma	3,846	51.8	(49.6–53.9)	1,808	59.8	(54.4–65.0)	2,038	46.9	(44.3–49.4)
Tennessee	4,771	53.8	(51.7–55.9)	2,343	63.3	(56.9–69.3)	2,428	47.3	(44.7–50.0)
Utah	5,997	38.8	(37.3–40.2)	1,854	49.6	(45.3–54.0)	4,143	34.8	(33.1–36.5)
Virginia	7,045	55.2	(53.6–56.8)	2,859	67.9	(64.0–71.6)	4,186	49.1	(47.2–51.0)
Washington	9,918	49.0	(47.6–50.4)	3,888	60.2	(56.4–63.9)	6,030	42.6	(42.0–45.2)
West Virginia	5,578	43.4	(41.8–45.1)	2,619	56.5	(52.8–60.2)	2,959	35.9	(34.0–37.9)
Wisconsin	5,360	44.3	(42.1–46.5)	2,174	60.1	(54.0–65.9)	3,186	37.0	(34.5–39.7)
Puerto Rico	5,783	73.4	(71.8–74.9)	2,896	80.0	(76.6–83.0)	2,887	70.0	(67.9–72.0)

**Abbreviations:** CI = confidence interval; DC = District of Columbia.

**TABLE 2:** Age-adjusted percentage of adults aged ≥18 years who reported being advised by a health professional to reduce dietary sodium intake, by hypertension status — 26 states, the District of Columbia, and Puerto Rico, Behavioral Risk Factor Surveillance System, 2013

	Overall			Self-reported hypertension			No self-reported hypertension		
			
State/Area	No.	%	(95% CI)	No.	%	(95% CI)	No.	%	(95% CI)
Arkansas	4,475	22.6	(20.8–24.4)	2,225	44.5	(39.6–49.5)	2,250	10.7	(9.1–12.6)
Connecticut	6,551	21.7	(20.2–23.2)	2,586	49.9	(44.9–55.0)	3,965	10.9	(9.5–12.5)
DC	3,996	27.4	(25.3–29.6)	1,622	60.7	(53.8–67.2)	2,374	13.3	(11.4–15.5)
Hawaii	6,977	24.3	(22.8–25.8)	2,195	49.1	(44.6–53.7)	4,782	14.8	(13.4–16.4)
Indiana	4,360	20.5	(19.1–22.0)	1,898	40.5	(36.3–44.9)	2,462	9.8	(8.5–11.3)
Iowa	7,186	17.8	(16.7–19.0)	2,874	40.4	(36.2–44.7)	4,312	8.9	(7.9–10.1)
Kansas	10,932	17.0	(16.2–17.8)	4,428	37.1	(34.0–40.3)	6,504	7.9	(7.2–8.7)
Kentucky	9,677	28.2	(26.9–29.5)	4,689	60.3	(56.5–63.9)	4,988	10.1	(9.0–11.3)
Maine	4,490	19.3	(17.8–20.8)	1,794	45.8	(39.7–52.0)	2,696	9.1	(7.9–10.6)
Maryland	11,489	23.8	(22.6–25.0)	4,898	48.5	(44.7–52.4)	6,591	12.4	(11.2–13.6)
Massachusetts	3,323	18.1	(16.1–20.3)	1,336	37.8	(31.4–44.7)	1,987	9.2	(7.4–11.5)
Minnesota	12,398	13.5	(12.4–14.6)	4,249	31.7	(28.0–35.7)	8,149	7.0	(6.0–8.2)
Mississippi	6,610	27.0	(25.5–28.5)	3,503	49.3	(45.0–53.5)	3,107	12.6	(11.1–14.2)
Missouri	5,457	19.8	(18.3–21.4)	2,502	45.0	(39.2–51.0)	2,955	9.9	(8.5–11.4)
Montana	4,508	13.8	(12.5–15.1)	1,693	33.3	(28.1–38.8)	2,815	6.2	(5.2–7.3)
Nebraska	7,660	17.1	(15.9–18.3)	3,086	35.1	(31.0–39.5)	4,574	8.5	(7.4–9.7)
New Jersey	3,715	23.5	(21.6–25.5)	1,359	50.5	(42.8–58.2)	2,356	12.5	(10.7–14.6)
North Carolina	3,808	24.1	(22.4–25.8)	1,735	47.1	(41.9–52.4)	2,073	12.3	(10.7–14.0)
North Dakota	6,941	15.1	(14.1–16.2)	2,569	36.6	(31.9–41.5)	4,372	6.5	(5.6–7.5)
Ohio	7,160	20.2	(18.9–21.5)	3,076	40.9	(37.0–44.9)	4,084	9.8	(8.6–11.2)
Oklahoma	3,835	22.6	(21.0–24.2)	1,798	40.3	(35.4–45.4)	2,037	12.4	(10.9–14.2)
Tennessee	4,756	23.2	(21.5–25.0)	2,329	41.9	(37.9–46.0)	2,427	11.5	(9.9–13.3)
Utah	5,988	14.5	(13.5–15.6)	1,842	35.5	(31.3–39.8)	4,146	6.7	(5.9–7.7)
Virginia	7,065	22.6	(21.4–23.9)	2,857	48.8	(45.0–52.6)	4,208	10.1	(9.0–11.2)
Washington	9,926	17.7	(16.7–18.7)	3,871	40.3	(36.6–44.1)	6,055	8.3	(7.4–9.2)
West Virginia	5,557	22.4	(21.1–23.6)	2,597	43.1	(39.4–46.9)	2,960	9.8	(8.7–11.0)
Wisconsin	5,350	18.6	(16.9–20.4)	2,169	41.4	(35.6–47.3)	3,181	8.6	(7.0–10.6)
Puerto Rico	5,781	41.4	(39.7–43.0)	2,896	61.6	(57.9–65.1)	2,885	27.2	(25.2–29.2)

**Abbreviations:** CI = confidence interval; DC = District of Columbia.

## Footnotes

* South Census region includes Alabama, Arkansas, Delaware, District of Columbia, Florida, Georgia, Kentucky, Louisiana, Maryland, Mississippi, North Carolina, Oklahoma, South Carolina, Tennessee, Texas, Virginia, and West Virginia.

† Available at http://www.nyc.gov/html/doh/html/pr2013/pr008-13.shtml.
